# Getting to the heart of Carotid and Vertebral imaging in acute ischemic stroke: an all-encompassing cross-sectional comparative analysis of Colour Doppler Ultrasound, CT Angiography, and MR Angiography

**DOI:** 10.1093/bjro/tzaf031

**Published:** 2025-12-17

**Authors:** Komal Verma Saluja, Mahesh Kumar Swami, Drishya Pillai, Manisha Meena, Dharm Raj Meena

**Affiliations:** Department of Radiology, Government Medical College, Kota 324005, Rajasthan, India; Department of Radiology, Government Medical College, Kota 324005, Rajasthan, India; Department of Medicine, Meditrina Hospital, Palakkad 678006, Kerala, India; Department of Radiology, Government Medical College, Kota 324005, Rajasthan, India; Department of Radiology, Government Medical College, Kota 324005, Rajasthan, India

**Keywords:** ischaemic stroke, carotid atherosclerosis, carotid stenosis, vulnerable plaques, vertebral stenosis, colour doppler ultrasound, stroke imaging protocol, CT angiography, MR angiography

## Abstract

**Objectives:**

This study presents a comprehensive comparison of minimally-invasive extracranial neck imaging modalities—Colour Doppler ultrasound (CDUS), CT angiography (CTA), and MR angiography (MRA)—in acute ischaemic stroke (AIS) patients. The aim was to evaluate vessel stenosis, its related parameters, and assess the role of early CTA/MRA in AIS.

**Methods:**

Categorical and continuous data were compared with Chi-square and independent Sample *t*-test, respectively. Spearman rank correlation matrix was performed for non-linear CDUS variables. The agreement between various imaging modalities was calculated with kappa (*k*) coefficient.

**Results:**

AIS was most common in males, aged 61-70 years, associated with hypertension and smoking (*P*-value < .05). Seventy-four plaques were identified in 50 patients, with good agreement between the 3 imaging (*k* > 0.6). CDUS was limited in evaluating Vertebral Arteries and plaque characterization. CTA/MRA showed higher sensitivity for defining stenosis and plaques, with good-excellent agreement between them (*k* > 0.6). CTA and MRA identified 40 and 43 vulnerable plaques, respectively.

**Conclusions:**

Colour Doppler ultrasound is subjective, comprehensive assessment of anatomic and hemodynamic parameters but lacks sensitivity in identifying vulnerable plaques. CTA/MRA have better sensitivity with good soft tissue differentiation especially in lesser stenosed vessels.

**Advances in knowledge:**

Our results support preferred use of MRA/CTA as first-line modalities in time-sensitive scenarios like acute stroke and need to move beyond CDUS-based assessment. These show promise in detecting vulnerable plaque and predicting AIS risk/recurrence; in patient triage, and to guide early intensive treatment. Longitudinal studies are required to assess risk reduction by early advanced imaging.

## Introduction

Cerebral ischaemic stroke (IS) affects around 12 million people annually, making it one of the leading causes of disability, dementia, and death.^1^^,^^2^ Cerebral infarction due to carotid atherosclerotic disease accounts for 80% of strokes^3^ and is thus one of the major preventable causes.^4^

Patients with diabetes, asymptomatic carotid plaques and/or carotid stenosis, have increased risk of large artery atherosclerosis.^5^ Carotid artery stenosis (CAS) comprises 18%-25% of IS cases, while vertebral artery stenosis (VAS) accounts for 20%-25% of posterior circulation strokes.^5^ Three in ten IS survivors report recurrence.^6^ Thus, a crucial part of acute ischaemic stroke (AIS) evaluation includes carotid/vertebral imaging for guiding initial management and secondary prevention.^7^

Conventional angiography, although the gold standard for detecting carotid stenosis, has now been largely replaced by non- or minimally-invasive modalities like Colour Doppler ultrasound (CDUS), CT angiography (CTA), and MR angiography (MRA).^8^

Traditionally, CDUS has been the screening test of choice in AIS but caution is exercised to avoid it as a sole diagnostic test preoperatively.^9^ CT angiography or MRA are usually done in cases with dubious results or cases planned for intervention.^10^ Currently, the decision for intervention (endarterectomy or stenting) is still primarily based on the extent of luminal stenosis.^5^^,^^11^

However, the role of plaque characteristics to predict IS has gained attention.^10^ Morphological features like intraplaque haemorrhage (IPH), plaque ulceration/neovascularity, thin/ruptured fibrous cap (FC), and lipid rich necrotic core (LRNC) have increasingly been associated with heightened risk of stroke.^4^^,^^10^ Hence, in times of such shifting paradigms, utility of CTA/MRA in AIS needs to be redefined.

With our study, we explore whether image selection should be driven by preconceived treatment plans, which primarily focus on stenosis alone or prefer early advanced imaging in all AIS cases to enable a holistic vessel assessment and then tailor the treatment.

To the best of our knowledge, no prior study has compared CDUS, CTA, and MRA, in this population specifically in AIS cases, where rapid and accurate vascular imaging is critical.^12^^,^^13^ The objectives were to assess the correlation between clinical history, risk factors, and imaging; to assess diagnostic accuracy of CDUS, CTA, MRA; to assess the possible utility of MRA/CTA as first-line investigations in AIS. A sub-study to evaluate the correlation between various DUS parameters was also done.

## Methods

This is an observational, cross-sectional study, done between December 2022 and November 2023, conducted in a tertiary care hospital. Patients with history and clinical findings of AIS were selected using purposive sampling technique without any age, sex, or ethnic discrimination. The sample size was 50, who were selected in sequence from Medicine/Neurology departments. A detailed history, thorough physical examination, and risk factors (hypertension, diabetes mellitus, smoking, and ischaemic heart disease) were documented. Patients with haemorrhagic stroke, rheumatic heart disease or intra cardiac clots, vasculitis/connective tissue disorders, on statin therapy were excluded; as were patients with head injury or those having primary/metastatic brain tumours. The study protocol was approved by the Institutional Ethics Committee and written informed consent was obtained from the patients.

### Data collection and equipment

Colour Doppler ultrasound was done using Sonoscape P20 Machine. Bilateral common carotid (CCA), external carotid (ECA), internal carotid (ICA), and vertebral arteries (VA) were assessed. Peak systolic velocity (PSV), end-diastolic velocity (EDV), resistive index (RI), and velocity ratio between ICA and CCA were included. Plaque characteristics, spectral broadening, and degree of stenosis were noted. Based on echogenicity, CDUS classified plaques as Types I-V. B-mode was used for imaging atherosclerotic plaques and intima-media thickness (IMT), while colour Doppler was used to visualize vascular lesions and flow abnormalities. All the examinations were performed at an insonation angle of 60°, with 13-6 MHz linear-array probe.

Bright Speed 16 Slice CT Scanner (GE HealthCare, Chicago, Illinois, United States) with Iohexol contrast was used for CT imaging. Plaque characterization, degree of stenosis of all the 8 vessels, and CT brain findings were gathered. These characteristics were also assessed using MRA with Philips 1.5 T Scanner used for non-contrast MRI scanning.

Each imaging was performed by separate dedicated radiologists, with a minimum of 10 years’ experience in neuroimaging, blinded from the findings of other imaging procedures.

### Study definitions

Plaque morphology is divided into 5 categories by CDUS, based on its echolucency—uniformly echolucent (1), predominantly echolucent plaques with less than 50% echogenic areas (2), predominantly echogenic plaques with less than 50% echolucent areas (3), uniformly echogenic plaques (4), and unclassifiable plaques due to heavy calcification and acoustic shadowing (5).^14^

Intima-media thickness was calculated in CCA and ICA between intimal and adventitial interface. Normal IMT is less than 0.8 mm. Intima-media thickness value of 0.8-1.0 mm has been considered indeterminate and ≥1.1 mm has abnormal.^15^

Normal PSV in ICA and CCA is 45-125 cm/s. Internal carotid PSV of ≥2.3 m/s and/or EDV ≥ 1.8 m/s, and/or ICA/CCA PSV ratio ≥ 4.0 indicate the presence of a 70% or greater narrowing of the diameter.^16^^,^^17^ Resistive index, a ratio that quantifies the resistance to blood flow in a vascular bed, is normally <0.7. It is calculated as the difference between PSV and EDV, divided by PSV.^18^

CT angiography classified plaques as fatty, mixed, and calcified based on Hounsfield density. CT angiography assessed luminal surface of plaques as smooth, irregular, or ulcerated; while MRA identified plaque characteristics like LRNC, IPH, thin or ruptured FC, ulcerated surface, and calcifications. All features except calcifications were considered markers of a vulnerable plaque.^19^ Artery stenosis was graded as mild (<50%), moderate (50%-69%), severe (70%-99%), or occlusion (100%), by both CTA and MRA.^20^

### Statistics and data analysis

The categorical and continuous data were expressed in terms of frequency (percentage) and mean (standard deviation), respectively. The association between categorical and continuous data was evaluated with Chi-square and independent sample *t*-test respectively. Spearman rank correlation matrix was performed for all the CDUS variables not linearly dependent on one another. The agreement between various imaging modalities was calculated with kappa (*k*) coefficient, and interpretated as poor (*k* < 0.2), fair (*k* = 0.21-0.40), moderate (*k* = 0.41-0.60), good (*k* = 0.61-0.80), and excellent (*k* = 0.81-1.0).^21^ Statistical analyses was performed with SPSS (IBM, Armonk, NY, United States) version 23.0 for Windows. A 2-tailed probability value (*P*-value) of <.05 was regarded as statistically significant.

## Results

### Baseline analysis—Demography

**Figure 1.**
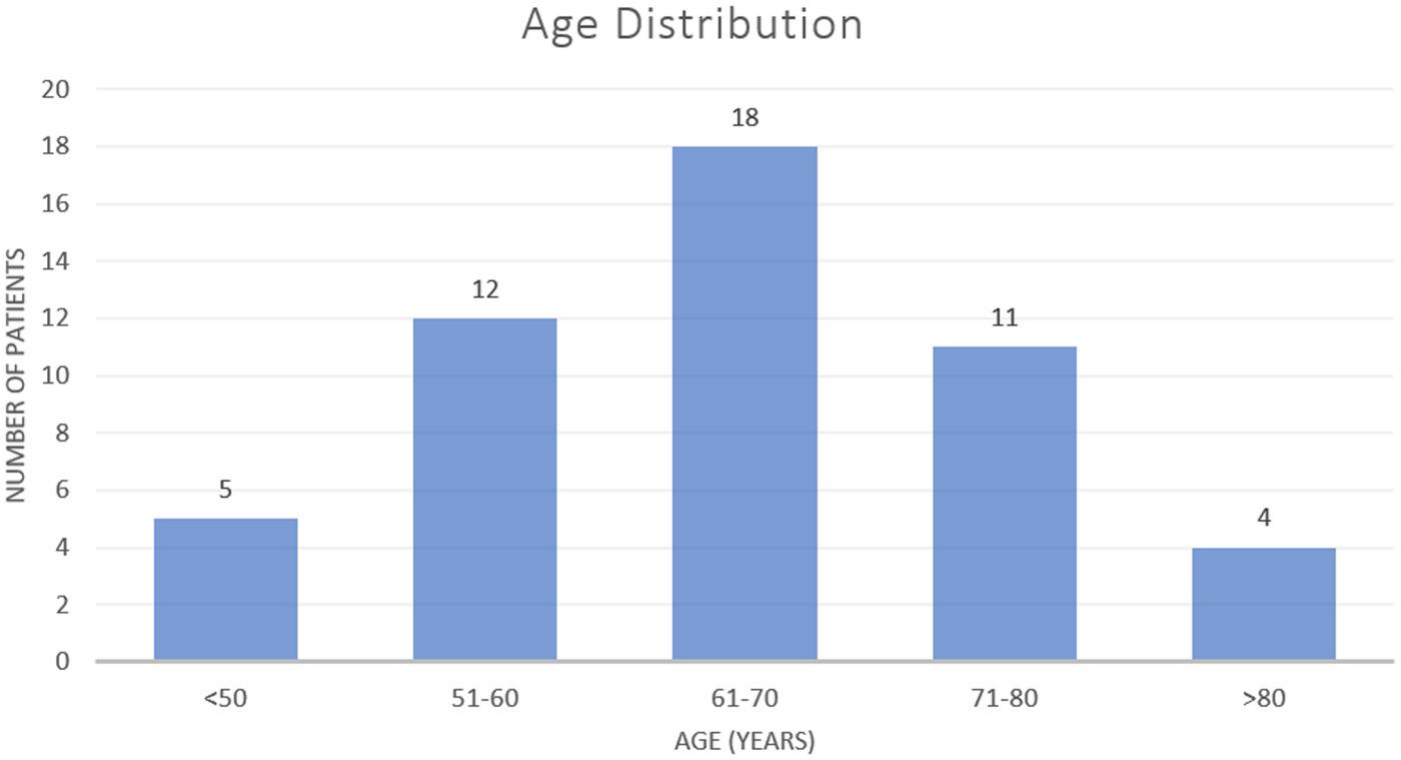
Bar graph elaborating the age distribution of the dataset. *x*-axis—age groups (in years); *y*-axis—number of patients (denoted by the height of each bar). The number is indicated on top of each bar as well.

The mean age of patients was 63.60 ± 13.34 years (range: 28-88 years), and 60% (30) of the patients were males. Figure 1 highlights the different age groups and the prevalence of stroke in each. The most affected age group was 61-70 years (36%). Among risk factors, highest prevalence was of hypertension and smoking (54% each) (Figure 1, Table 1).

**Table 1.** Baseline characteristics of the dataset (first column); including mean age, gender, and risk factors for stroke.

Age (mean ± SD)	63.60 years ± 13.34
Gender: Male	30 (60%)
Risk factors: prevalence (%)	
HTN	54%
DM2	12%
MI	0%
Smoking	54%

The second column has the corresponding value for each variable. The parantheses in second row, second column indicates their percentage of the total sample size.

### CDUS—a comprehensive analysis

The results and discussion would mention vessels in the order of CCA, ICA, ECA, and VA. Only the positive findings are being mentioned in text.

#### Plaques

**Figure 2.**
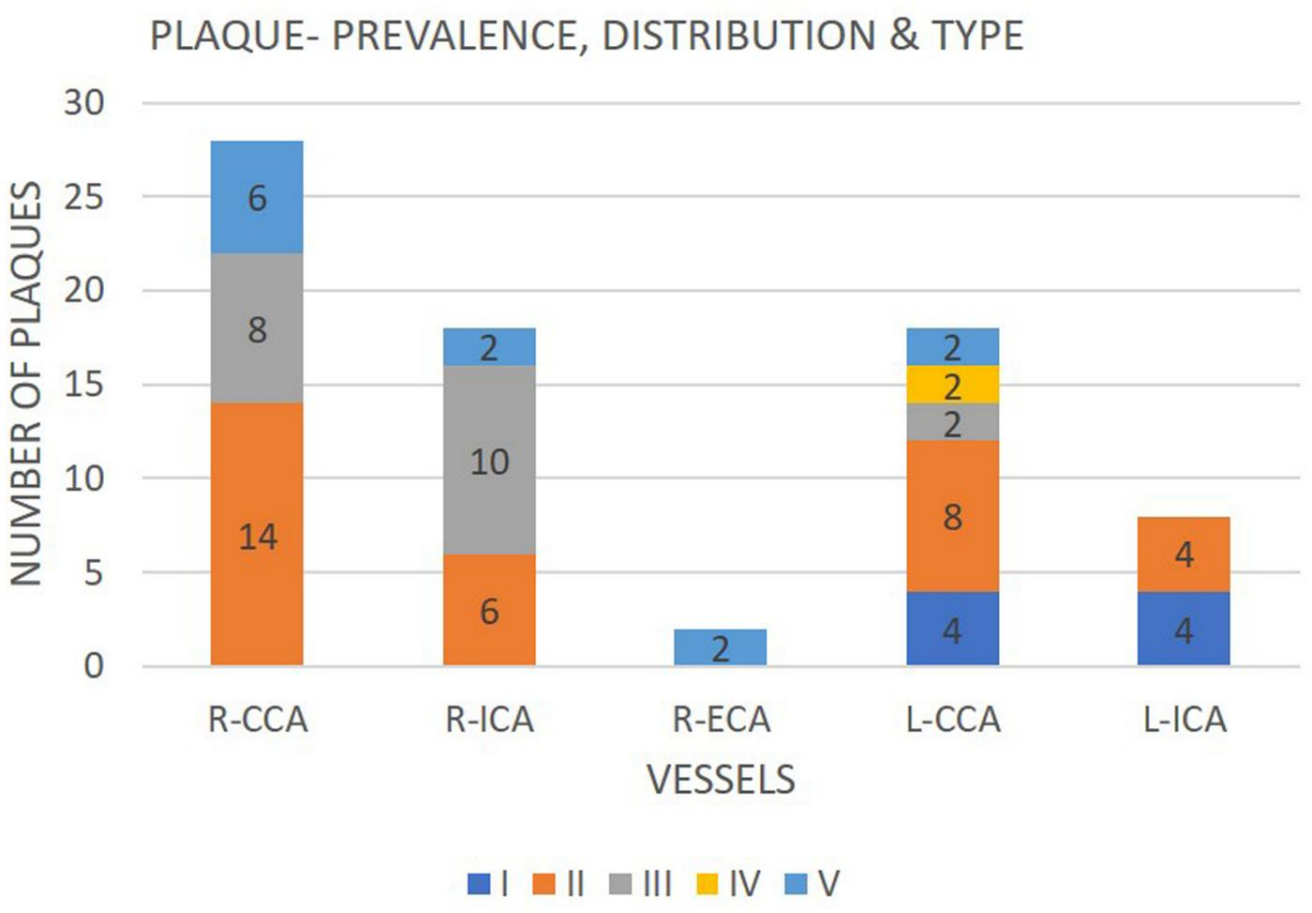
Clustered bar graph of the distribution, prevalence, and type of plaques. *x*-axis—5 bars, each for the vessels evaluated. Each bar has been divided into coloured sections, denoting the frequency and type of plaque, I-V. The colour attributed to the plaques have been mentioned at the bottom of the figure. The height of the bar indicates the total number of plaques detected in each vessel. R-VA, L-ECA, L-VA are not included as there were no plaques detected.

A total of 74 plaques were observed in all vessels, except left ECA and both VA. Type II (43.24%) and type III (27.03%) plaques were most common (Figure 2). R-CCA bulb involvement was noted in 14 cases (28%) and L-CCA bulb in 10 cases (20%).

Mean IMT was below 1 mm across all vessels. However, when stratified by the presence of plaque, the mean IMT in vessels with plaque was elevated (>1 mm), except R-ICA. Statistically significant increase was in L-ICA (*P*-value .001) (Table 2).

**Table 2.** Intima-media thickness and association with plaque.

Vessel	Plaque prevalence (*N*)	Mean IMT (mm)	Mean IMT in vessels with plaque (mm)	Mean IMT in vessels without plaque (mm)	*P*-value
R-CCA	28	0.95	1.00	0.89	.086
R-ICA	18	0.87	0.94	0.82	.066
L-CCA	18	0.95	1.02	0.91	.122
L-ICA	8	0.86	1.13	0.81	.001

The first column indicates the vessel evaluated. The second and third column has the corresponding vessels’ total plaque number and mean IMT, respectively. The fourth and fifth columns indicate the average IMT value (in mm) in the said vessel with and without plaque. The last column is the *P*-value between the fourth and fifth columns (assessed after the student *t*-test). *P*-value <.05 is statistically significant.

#### Velocities

**Figure 3.**
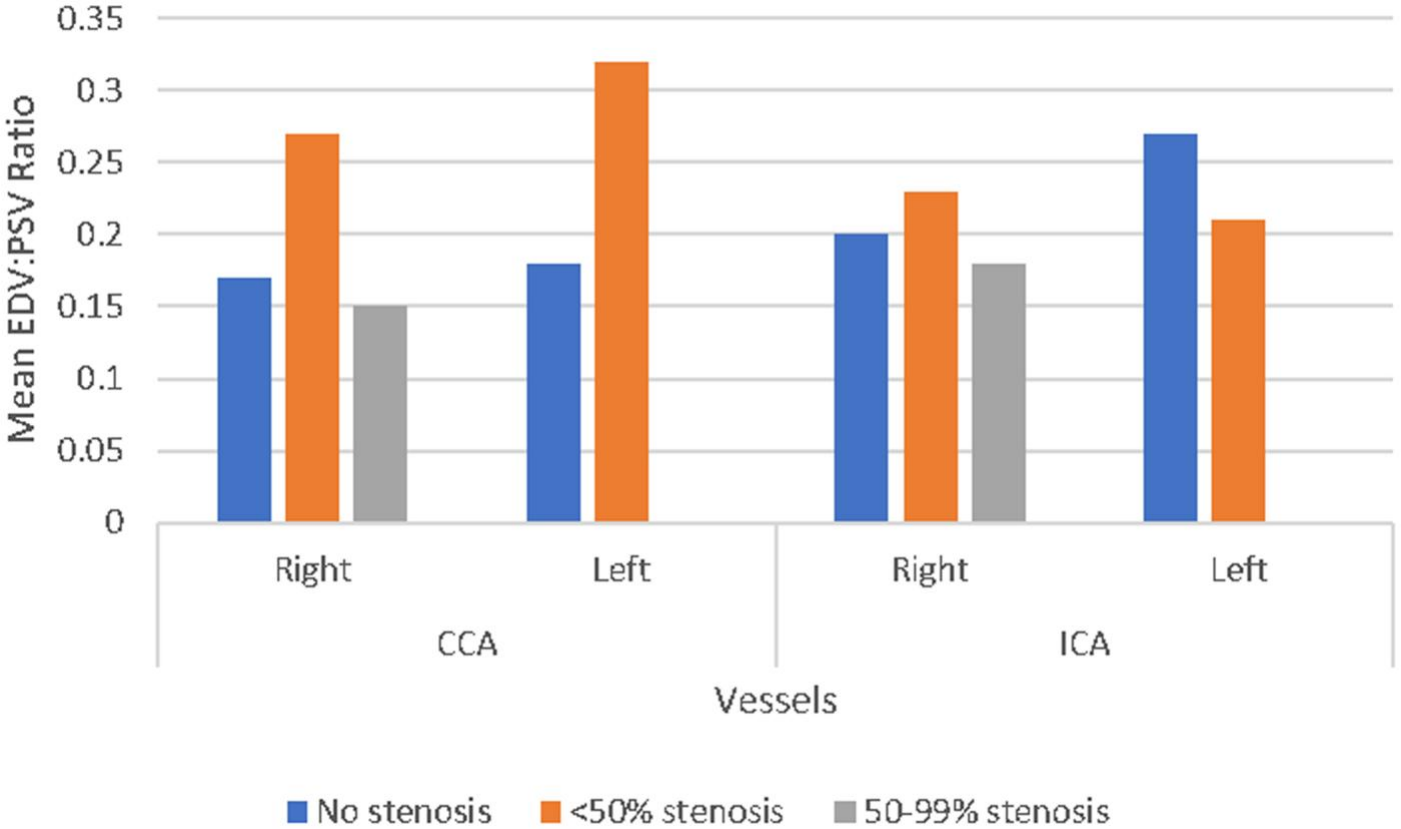
EDV:PSV ratio in CCA, ICA with varying stenosis. *x*-axis: right and left CCA, ICA. Grouped bars are present for each vessel, with blue denoting no stenosis, orange denoting <50% stenosis and gray denoting 50%-99% stenosis. The height of each bar indicates the mean EDV:PSV ratio. RI = 1 − (EDV/PSV).

Isolated PSV and EDV were non-conclusive. We found PSV ICA/CCA >1.8 in 6 patients and EDV ICA/CCA >2.4 in 8 cases. Resistive index, inversely proportional to EDV/PSV, was highest in right carotid arteries with 50-99% stenosis (Figure 3).

#### Correlation matrix

**Figure 4.**
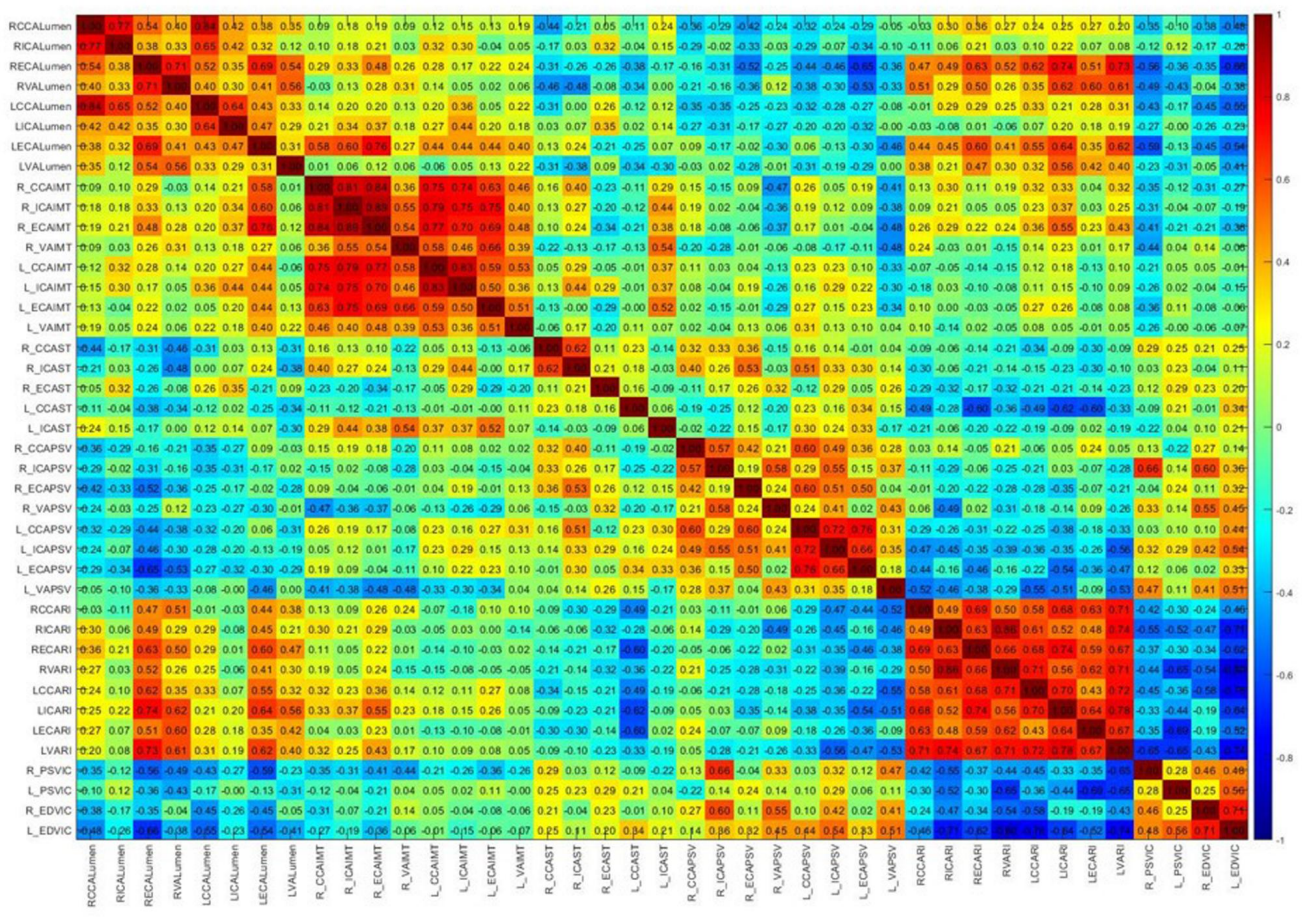
Correlation matrix between the variables obtained from CDUS, including lumen diameter, IMT, percentage stenosis, PSV, RI, and velocity ratio between ICA/CCA. The 3 vessels with no stenosis have been removed from the matrix. The cells are shaded according to strength of relationships based on the correlation coefficient, −1 to +1. The colour represents positive (red) or negative (blue) correlations and the magnitude of correlation coefficients, as indicated by the colour bar on the right, with darker shade corresponding to stronger correlation. Each cell has the *R*-value derived from the spearman correlation done between the variables on its corresponding abscissa and ordinate.

Spearman correlation matrix (Figure 4) found that *lumen diameters* were strongly and positively correlated with each other, notably of CCA and ICA. Diameters were inversely correlated with stenosis severity, except for a non-significant positive correlation between ICA diameter and ECA stenosis bilaterally. Lumen diameters showed negative correlation with PSV (statistically insignificant).

*Inter-vessel IMT* showed positive correlation, strongest between CCA and ICA. Intima-media thickness of ICA and CCA was strongly and positively correlated with ICA stenosis as well as their respective PSV.

*Vessel stenosis—*A strong negative correlation of left CCA stenosis was noted with RI in all vessels, including VA.

*Peak systolic velocity*—Lastly, a positive correlation amongst PSV of all vessels except VA was seen. A negative correlation between VA-PSV and RI of all vessels was noted.

### CT angiography and MR angiography

***Plaques**—*Fourteen arteries were devoid of any atherosclerotic plaques on both CTA and MRA. Interestingly 14 cases were found with no intracranial vessel anomaly by both MRA/CTA. M2 of right MCA was the most affected vessel amongst the remaining cases.

**Figure 5.**
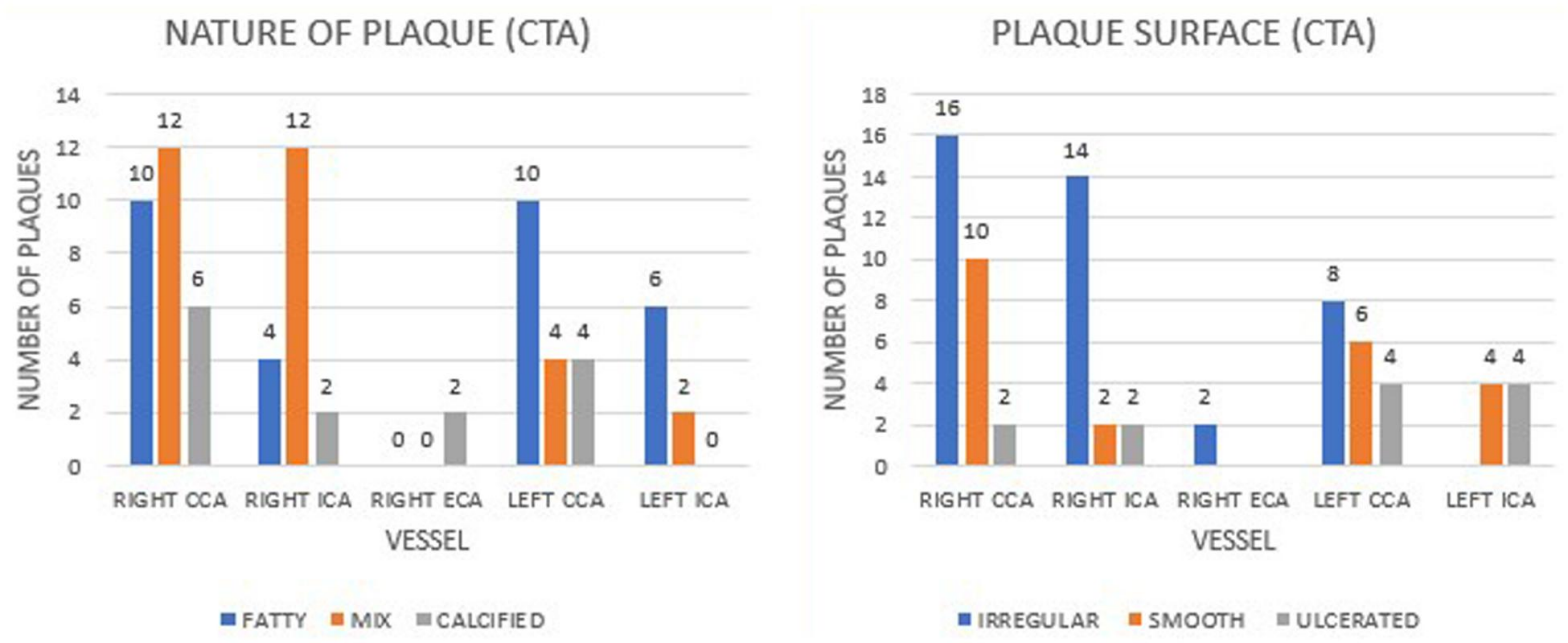
Grouped bar graphs indicating the plaque type (left panel) and the plaque surface (right panel)—as seen in CTA. The *x*-axis indicates the vessel assessed and *y*-axis denote the number of plaques; with the number being labelled on top of each bar. Left panel—the plaques are divided as fatty (blue), mixed (orange), and calcified (gray). Right panel—the plaque surface is of the subtypes—irregular (blue), smooth (orange), and ulcerated(gray). Again, R-VA, L-ECA, L-VA have not been mentioned as no plaques could be identified.

CT angiography showed a plaque distribution similar to CDUS. Fatty and mixed plaques were predominant [*n* = 30 (40.5%) each]. Plaque prevalence and their surface characteristics are elaborated in Figure 5. Highest plaque prevalence was in R-CCA. Four right and 2 left VAs were hypoplastic. Forty out of 74 detected plaques were vulnerable (irregular/ulcerated), 18 of these were in vessels with mild/moderate stenosis. Eleven out of 40 plaques were noted on the unaffected side; 9 of which had mild/moderate stenosis.

**Figure 6.**
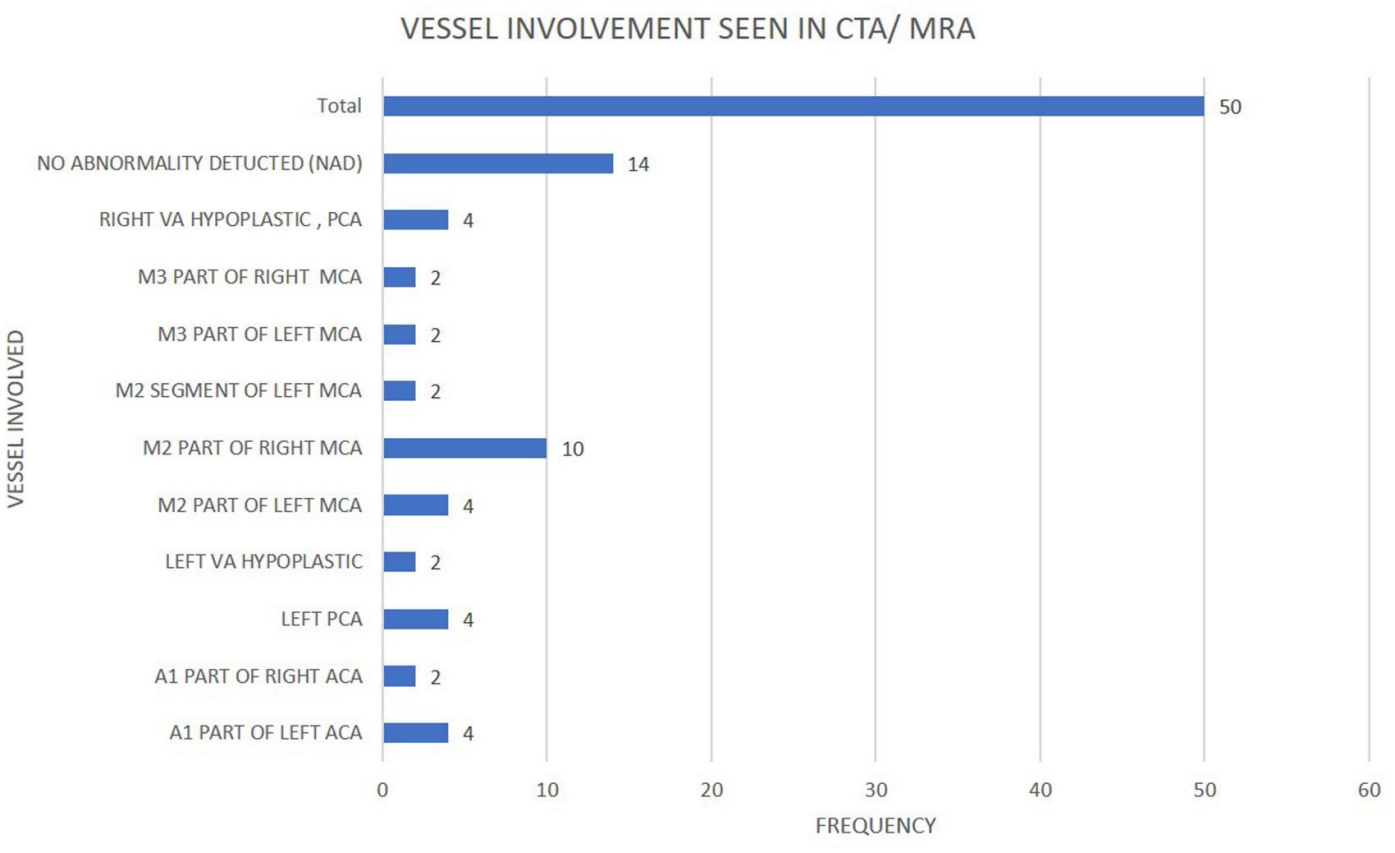
Bar graph denoting the various intracranial arteries affected in the AIS patients as seen on CTA and MRI. We have used a single figure for both imaging as the findings were same. Except for hypoplastic vessels, which have been mentioned in the figure itself, all the other vessels involved, were thrombosed in our dataset. Each horizontal bar has the corresponding frequency or number of cases.

Plaque prevalence and stenosis detected by MRA were similar to CTA but it identified 43 (vs 40 by CTA) vulnerable plaques in 28 patients: 21 patients of whom had intracranial vessel involvement. Of these 21 patients, bilateral, ipsilateral, and contralateral carotid involvement were seen in 4, 13, and 4 cases, respectively. Of the 7 patients with no intracranial vessel anomaly, 3 had bilateral carotid vulnerable plaques. A total of 14 patients (similar to CTA) had no cerebral vessel involvement. The vulnerable plaque features and distribution are demonstrated in Table 3. Interestingly, 33 out of the 43 vulnerable plaques (76%) were seen in arteries with mild to moderate stenosis. Maximum vulnerable plaques were seen in R-CCA. Figure 6 highlights the intracranial vessel distribution in CTA and MRA (both identical).

**Table 3.** Distribution and prevalence of vulnerable plaque characteristics across vessels.

	R-CCA	R-ICA	L-CCA	L-ICA
IPH	3	2	0	1
LRNC	16	10	10	2
Thin/ruptured FC	15	9	11	3
Erosion	3	3	2	3

The first column denotes the various vulnerable plaque characteristics, viz IPH, intra plaque haemorrhage; LRNC, lipid rich necrotic core; FC, fibrotic core. Second to fourth columns indicate vessels R-CCA, R-ICA, L-CCA, and L-ICA, respectively. Each cell under these columns indicates the number of corresponding plaque feature in the first column. These characteristics were found in isolation or in combination. Isolated LRNC was not considered as a vulnerable plaque.

#### Comparing CDUS, CTA, and MRA

Role of CDUS, CTA, and MRA in detecting arterial pathology is depicted in Table 4. Colour Doppler ultrasound identified more number of plaques where the stenosis was mild, but with >50% stenosis, MRA/CTA fared better. All the 3 had a moderate to good/excellent agreement with each other (Table 5).

**Table 4.** Role of CDUS, CTA, and MRA in detecting arterial pathology.

Stenosis	CDUS—number of plaques	CTA—number of plaques	MRA—number of plaques
16%-49%			
Right CCA	19	19	16
Right ICA	12	10	8
Right ECA	2	2	2
Left CCA	14	15	15
Left ICA	5	5	4
50%-69%			
Right CCA	4	3	7
Right ICA	0	0	2
Left CCA	2	2	2
Left ICA	1	1	2
70%-99%			
Right CCA	4	5	4
Right ICA	2	2	2
Left CCA	2	1	1
Left ICA	1	0	1
100%			
Right CCA	1	1	1
Right ICA	4	6	6
Left ICA	1	2	1

The first column denotes the vessel and are divided under 4 sections, depending on the degree of stenosis (in %). The vessels with no pathology have not been mentioned. The second, third, and fourth columns indicate the number of plaques detected by CDUS, CTA, and MRA, respectively. Each cell under these columns indicates the number of plaques corresponding to each vessel in the first column. The % in first column indicates the degree of stenosis.

**Table 5.** Agreement between the 3 tested imaging modalities, that is, CDUS, CTA, MRA in bilateral ICA and CCA.

Vessel	CDUS vs CTA	CDUS vs MRA	CTA vs MRA
R-CCA	Good (*k* = 0.785, *P*-value < .0001)	Good (*k* = 0.678, *P*-value < .0001)	Good (*k* = 0.744, *P*-value < .0001)
R-ICA	Good (*k* = 0.795, *P*-value < .0001)	Good (*k* = 0.640, *P*-value < .0001)	Excellent (*k* = 0.824, *P*-value < .0001)
L-CCA	Good (*k* = 0.667, *P*-value < .0001)	Good (*k* = 0.667, *P*-value < .0001)	Good (*k* = 0.617, *P*-value < .0001)
L-ICA	Good (*k* = 0.778, *P*-value < .0001)	Good (*k* = 0.800, *P*-value < .0001)	Moderate (*k* = 0.600, *P*-value = .005)

The first column indicates the 4 vessels, namely right and left ICA and CCA. The second, third, and fourth columns are agreement analysis between sets of 2 modalities, CDUS vs CTA, CDUS vs MRA, and CTA vs MRA, respectively. Each column shows the degree of agreement, along with the *k* and *P*-values in the parentheses.

## Discussion

The rising burden of CVA is driven by key risk factors such as hypertension, obesity, hyperglycaemia, ambient pollution, and smoking. Elevated systolic blood pressure is the single most important risk factor for stroke.^22^

Our results corroborate the strong association of hypertension and smoking (Figure 1, Table 1). Higher predisposition of males is well documented, and was confirmed in our study.^23^ The susceptible age group is predominantly 51-70 years.^23^ Our study showed 61-70 years as the most affected.

While type I and II plaques are typically seen in symptomatic patients, our study found type II and III to be the most common (Figure 2). Patients with echolucent carotid plaques are at three times higher risk of recurrence.^24^ However, CDUS was unable to distinguish IPH from a LRNC.

The site of plaque is equally vital. The CCA bulb is susceptible for atheromatous changes due to turbulent blood flow and low shear stress. Carotid bulb plaques can be more predictive than vessel plaques.^24^ Our study showed 10 bilateral bulb involvement—less prevalent than vessel plaque, but assessing them as predictors of recurrence would require longitudinal assessment.

Colour Doppler ultrasound of VA is challenging. Technical difficulties, their deep and posterior origin, the presence of calcified lesions, tortuous course, or short neck stature reduce the sensitivity.^14^ Unlike CAS, consensus ultrasound grading criteria for VAS is lacking.^14^ In such cases, MRA/CTA can be used as a primary imaging. In our study, CTA/MRA identified 6 cases of hypoplastic VA, but no VA stenosis. Such congenital vertebral pathologies can also result in AIS.^14^^,^^25^

Intima-media thickness is reflective of either an early atherosclerotic stage or vascular remodelling, causing smooth vessel hyperplasia.^26^ However, its credibility as an independent predictor of AIS is questionable due to limited methodologic standardization and unclear end points.^8^^,^^26^ Compared to IMT, carotid plaque characteristics may serve as better predictors of recurrent vascular events.^27^^,^^28^ We noted raised mean IMT values in vessels with plaque. No significant association of AIS with increased IMT was established in this study.

Raised PSV indicates significant stenosis or turbulent blood flow, thus increased risk of AIS.^29^ Several pitfalls while measuring these velocities are tandem occlusion, contralateral carotid, or vertebral occlusion, elongated or high grade stenoses, proximal CCA occlusions, and varying cardiac output or aortic valve lesions, leading to diagnostic uncertainty.^14^ ICA/CCA PSV or ICA/CCA EDV ratios increase accuracy by accounting for individual variations in cardiac output and other confounding factors.^30^ ICA/CCA PSV and RI of our study were associated with vessel atherosclerosis.

Resistive index quantifies elasticity and compliance of vessel walls. Elevated RI implies an increased vascular resistance. The RI in CCA increases in patients with carotid bulb atherosclerosis and with age.^31^ In our study, we had the highest RI in R-CCA with 50%-99% stenosis (Figure 3). Vertebral arteries RI, however, decreases in patients with severe carotid artery disease, mainly by increased diastolic flow velocity. Vertebral arteries resistance is reduced to maintain cerebral perfusion with occluded or severely stenosed carotid arteries.^32^ This highlights the importance and difference of VA pathophysiology.

The spearman matrix assessed lumen diameter, degree of stenosis, IMT, and velocities (Figure 4). A strong positive correlation between inter-vessel lumen diameters could suggest generalized atherosclerosis. This also explains the positive correlation between degree of stenosis and IMT.

The negatively correlated ICA lumen diameter and ECA stenosis could indicate a redirected flow.

A negative correlation between lumen diameters and PSV, as well as a positive correlation between PSV and RI, is mathematically obvious. Strikingly, VA-PSV is negatively correlated with RI across all vessels. A strong negative correlation of left CCA stenosis is noted with RI in all vessels. Both suggest a compensatory hemodynamic response to anterior circulation stenosis. With increasing CCA stenosis, cerebral perfusion pressure decreases, prompting vasodilation in distal vascular beds, lowering RI values.^32^ Peak systolic velocity in all vessels are positively correlated with each other, except PSV in VA; again, possibly indicating the compensatory vasodilation.

The correlation between lumen diameter, degree of stenosis, IMT, and RI is all corroborative. An in-detail assessment of the entire correlation matrix is outside the scope of this article. This matrix underscores that although CDUS can assess multiple caveats of CAS, VAS, it is still limited by incongruence of variables and operator dependence.

## CTA and MRA-

CT angiography and MRA provide comprehensive assessment of intra and extracranial vasculature and identify their independent pathologies. This advantage is inherently absent in CDUS. Second, though considered to overestimate the atherosclerotic risk, they may in fact reveal the true disease burden and detect high-risk plaques irrespective of stenosis, thus improving the traditional stroke risk prediction.^33^ Stenosis endarterectomy even in patients with <50% stenosis may prevent recurrence if performed *early* after AIS *in such high-risk cases*.^34^

## Echoes, waves, or rays?

Colour Doppler ultrasound has been the screening test of choice with low cost, high temporal/spatial resolution, easy applicability, and no significant complications. Poor reproducibility, operator dependence, low panoramacity, low sensitivity in assessing VA or vessels with extensive calcification are its primary limitations.^14^ In our study, sensitivity to identify CAS by CDUS was at par (with CTA/MRA), in cases of mild stenosis but has limited ability to characterize the plaques, reducing its utility, especially in AIS.^14^

MR angiography has excellent tissue distinguishing properties, thus furnishes exquisite details of such plaques, with high diagnostic accuracy of IPH and LRNC.^11^^,^^34^ While vulnerable plaques are more accurately depicted by MRA, CTA is an excellent tool for identifying stenosis and certain vulnerable lesions. Lastly, there is minimal requirement of repeat acquisitions as they provide standardized dataset, which can be crosschecked by multiple observers (unlike CDUS).

**Figure 7.**
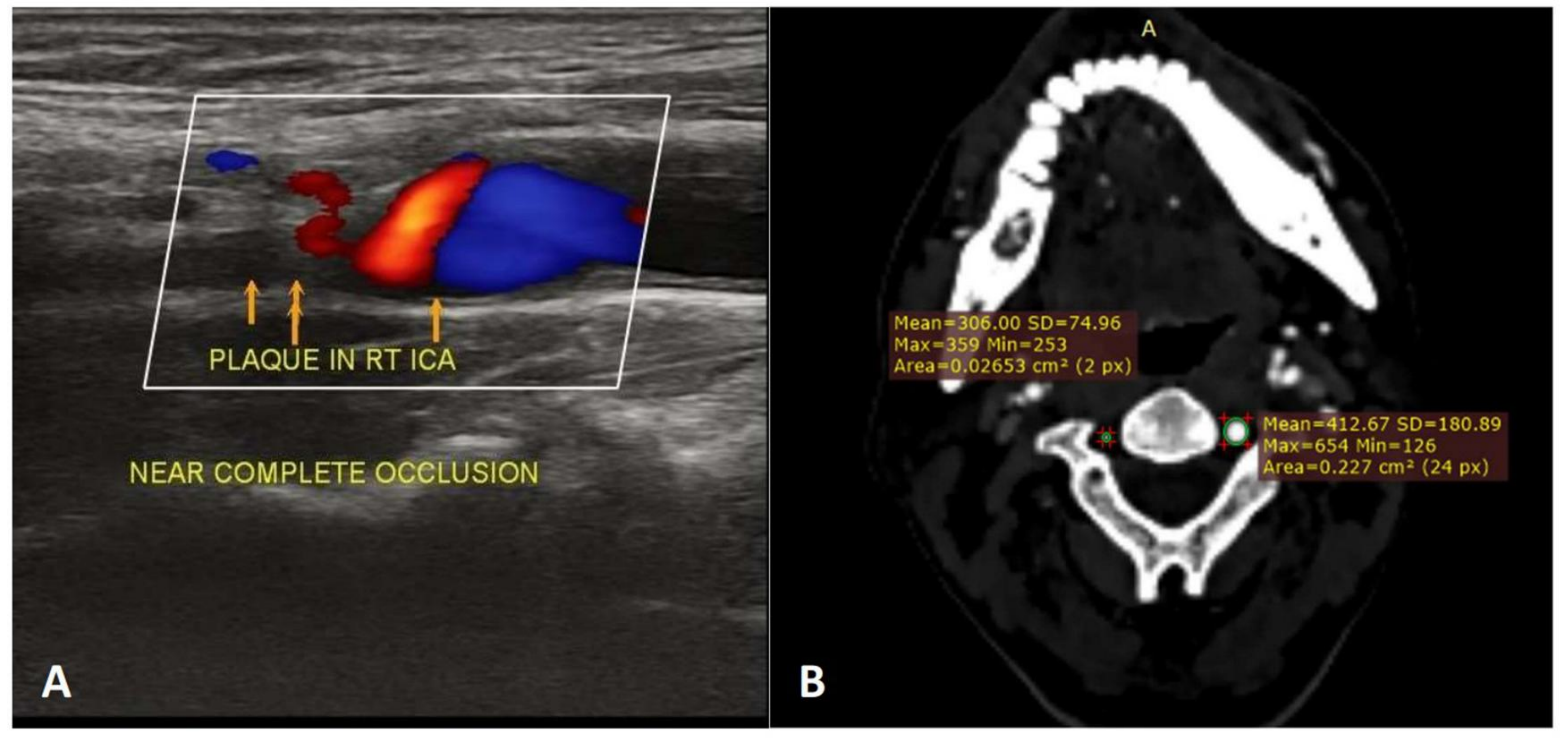
Neuroimaging findings in Case 1. (A) B-mode longitudinal scan CDUS at right carotid level shows plaque in right ICA causing complete occlusion with aliasing on CDUS; and (B) Axial CE-CT of neck vessels showing hypoplastic right VA as compared to normal left side.

**Figure 8.**
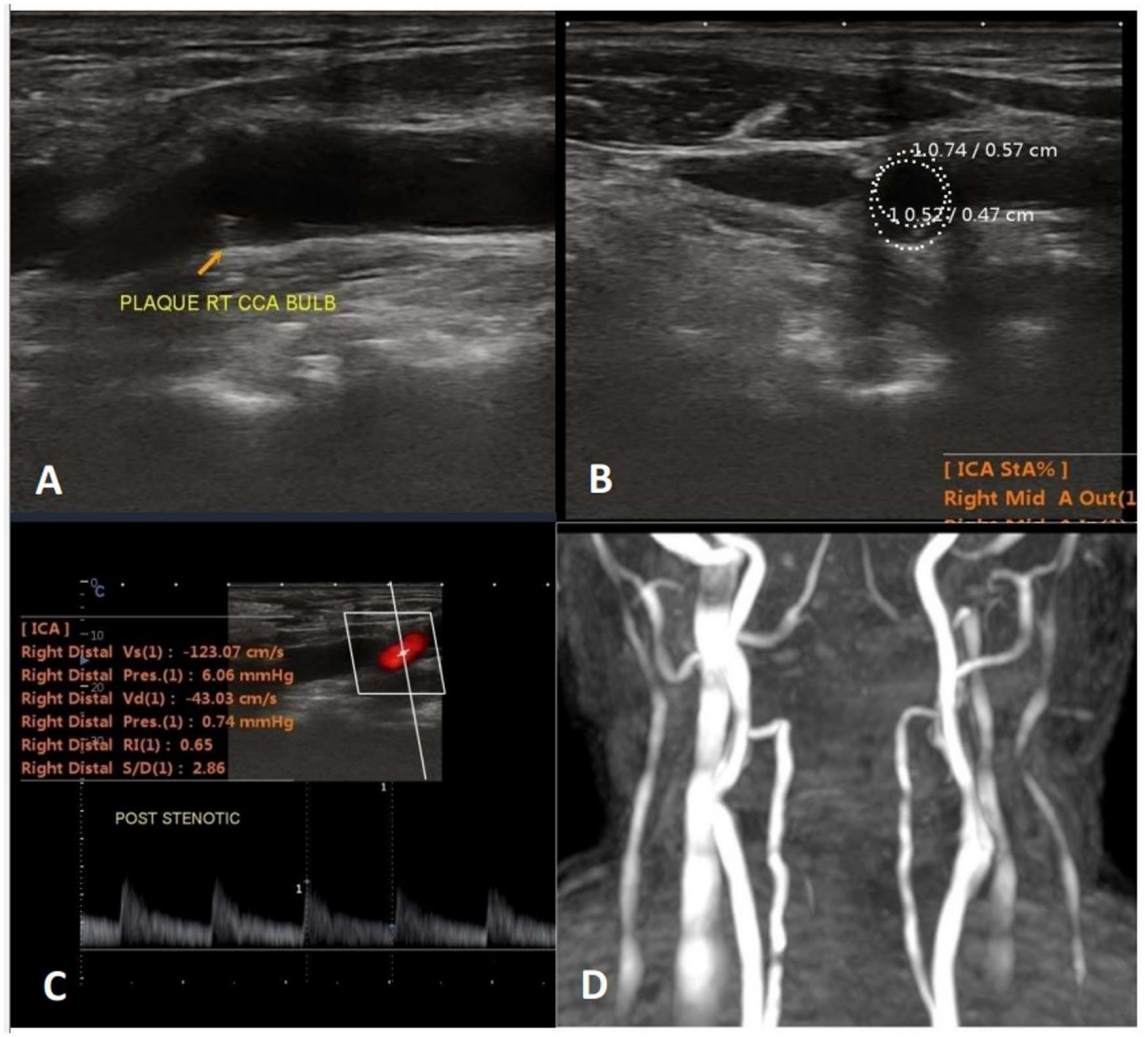
Neuroimaging findings in Case 2. B-mode longitudinal (A) and axial (B) scan of CDUS illustrating iso-hypo echoic plaque at right CCA bulb and extending in to ICA with increased IMT. Axial scan shows plaque causing 41% stenosis of right ICA lumen; (C) B-mode CDUS longitudinal scan at right ICA level shows increased PSV and spectral broadening; and (D) MRA shows normal carotid vessels on bilateral side.

**Figure 9.**
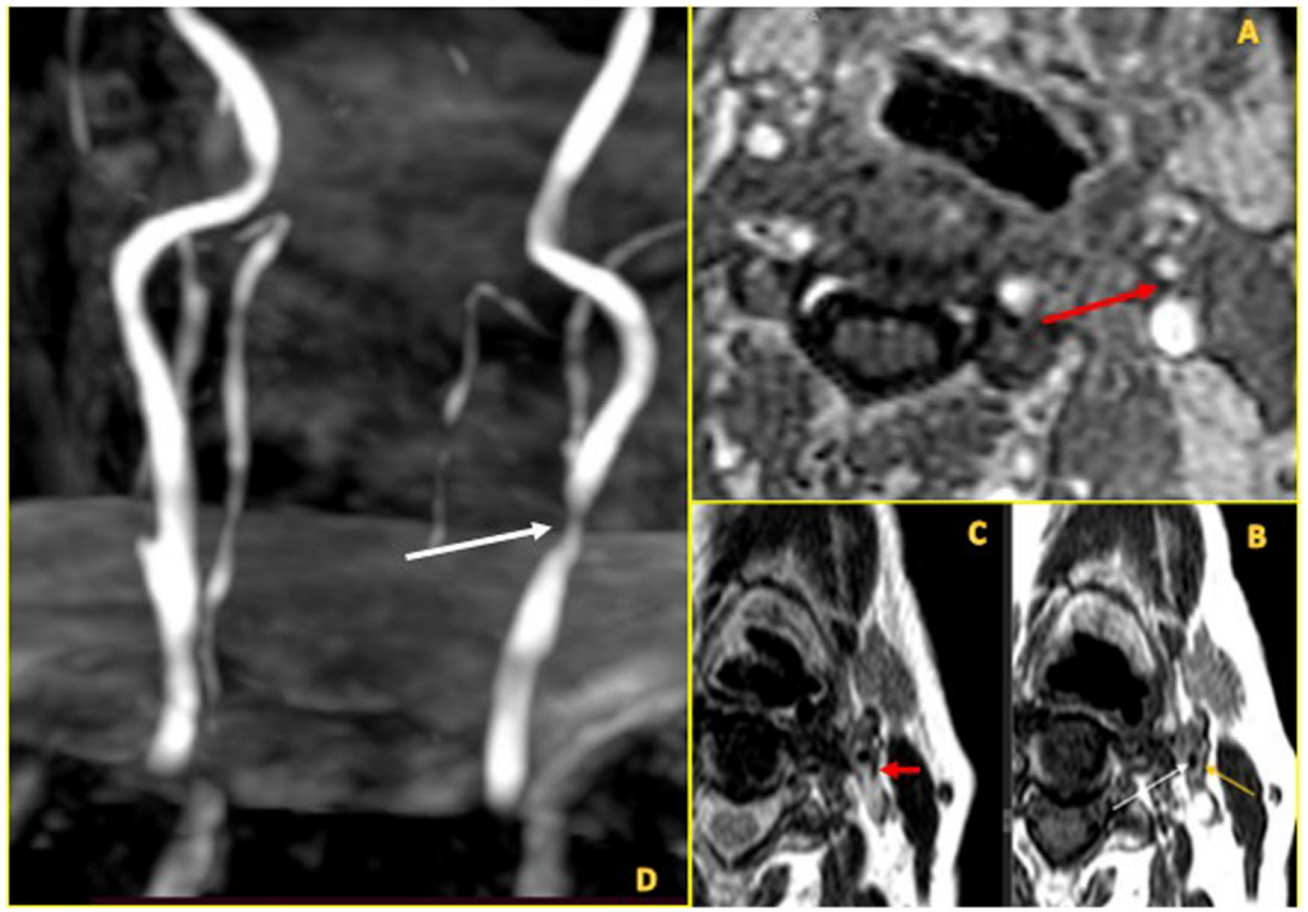
(A-D: clockwise from top right). (A) The PDFS sequence showing peripheral calcified low signal (marked by thick red arrow) with suppression of soft interior part on fat saturated images suggestive of fat. (B) Corresponding axial *T*1 weighted image demonstrates a peripherally calcified lesion, which is dark (solid thin yellow arrow) plaque with a soft interior (solid white arrow)—is suggestive of lipid rich plaque (lipid rich core). (C) Axial T2 showing peripheral calcified low signal marked by thick red arrow. (D) Maximum intensity projection (MIP) image shows significant narrowing of left CCA at bulb region (marked by thick white arrow).

On the downside, CTA has more radiation exposure; and MRA may have flow signal loss. The latter could be due to turbulent and slow flow, resulting in an inaccurate measurement of stenosis degree. Two cases have been depicted by the 3 imaging modalities in Figures 7 and 8 and Figure 9 depicts a vulnerable plaque by MRA.

Our study showed a good agreement between CDUS and CTA/MRA and an excellent agreement between CTA and MRA.^35^^,^^36^ CDUS was comparable to CTA/MRA to detect mild stenosis but was inferior in terms of moderate/severe stenosis. However, these results pertain to merely *identifying* stenosis. While expert-performed CDUS could rival CTA/MRA in accuracy, the similar expertise if applied with the excellent soft tissue imaging by MRA/CTA could benefit the patient by guiding timely intervention to prevent recurrence. A combination of imaging may be superior, but initial screening with MRA may help in early intervention, lesser repetitions, and reduced stroke recurrence.^4^^,^^36–38^

The strengths of our study are inclusion of AIS patients with varying degree of stenosis, utilizing experienced neuroradiologist, establishing agreement, and a comprehensive assessment between all 3 modalities.

The small sample size, lack of a control group, and the absence of a longitudinal follow-up are the primary limitations. A longitudinal study is quintessential to identify the causality as well as the outcome of early advanced imaging and intervention.

A longitudinal trial comparing CTA and MRA, its various new modalities, association of AIS with multiple elements of vulnerable plaques, time taken to surgery and the outcome are the future research questions of our project.

## Conclusion

All AIS cases need preliminary screening of extracranial vessels. Colour Doppler ultrasound done by an experienced radiologist is an outstanding multifaceted screening tool, but has its drawbacks in a time sensitive scenario of IS. Lumen stenosis should not be the sole surveillance and stratification tool. MRA/CTA offers superior assessment of stenosis and vulnerable plaques, with MRA providing a slight edge in plaque characterization. It may be time to reconsider guidelines and prioritize advanced imaging in AIS care. Despite the added time, cost, and radiation (in CTA), it could improve triage, enabling earlier intervention and enhanced patient outcome. The comparison of all 3 imaging in AIS, would be more informative if done on a larger multicentric dataset, with a control data.

## Data Availability

Data that support the findings of this study will be available from the corresponding author upon reasonable request.

## References

[1] Krishnamurthi RV , Ikeda T, Feigin VL. Global, regional, and country-specific burden of ischaemic stroke, intracerebral haemorrhage, and subarachnoid haemorrhage: a systematic analysis of the global burden of disease study 2017. Neuroepidemiology. 2020;54:171-179. 32079017 10.1159/000506396

[2] Feigin VL , Stark BA, Johnson CO, et al Global, regional, and national burden of stroke and its risk factors, 1990-2019: a systematic analysis for the Global Burden of Disease Study 2019. Lancet Neurol. 2021;20:795-820. 34487721 10.1016/S1474-4422(21)00252-0 PMC8443449

[3] Fernandes M , Keerthiraj B, Mahale AR, Kumar A, Dudekula A. Evaluation of carotid arteries in stroke patients using colour Doppler sonography: a prospective study conducted in a tertiary care hospital in South India. Int J Appl Basic Med Res. 2016;6:38-44. 26958521 10.4103/2229-516X.174007 PMC4765273

[4] U-King-Im JM , Young V, Gillard JH. Carotid-artery imaging in the diagnosis and management of patients at risk of stroke. Lancet Neurol. 2009;8:569-580. 19446276 10.1016/S1474-4422(09)70092-4

[5] Rothwell PM , Villagra R, Gibson R, Donders RC, Warlow CP. Evidence of a chronic systemic cause of instability of atherosclerotic plaques. Lancet. 2000;355:19-24. 10615886 10.1016/s0140-6736(99)04470-0

[6] Bluth E. Evaluation and characterization of carotid plaque. Semin Ultrasound CT MR. 1997;18:57-65. 9143066 10.1016/s0887-2171(97)90038-x

[7] Lim SN , Chang YJ, Lin SK. Extracranial carotid artery disease: risk factors and outcomes in patients with acute critical hemispheric ischemic stroke. J Ultrasound Med. 2016;35:341-348. 26764275 10.7863/ultra.15.03070

[8] O’Leary DH , Polak JF. Intima-media thickness: a tool for atherosclerosis imaging and event prediction. Am J Cardiol. 2002;90:L18-L21. 10.1016/s0002-9149(02)02957-0 12459422

[9] Cassola N , Baptista-Silva JC, Nakano LC, et al Duplex ultrasound for diagnosing symptomatic carotid stenosis in the extracranial segments. Cochrane Database Syst Rev. 2022;7:CD013172. 35815652 10.1002/14651858.CD013172.pub2 PMC9272405

[10] Dakis K , Nana P, Athanasios C, et al Carotid plaque vulnerability diagnosis by CTA versus MRA: a systematic review. Diagnostics (Basel). 2023;13:646. 36832133 10.3390/diagnostics13040646 PMC9955971

[11] Saxena A , Ng EY, Lim ST. Imaging modalities to diagnose carotid artery stenosis: progress and prospect. Biomed Eng Online. 2019;18:66-23. 31138235 10.1186/s12938-019-0685-7 PMC6537161

[12] Khaku AS , Tadi P. Cerebrovascular disease. In: StatPearls[Internet]. Treasure Island (FL): StatPearls Publishing; 2025. https://www.ncbi.nlm.nih.gov/books/NBK430927/

[13] Maroufi SF , Rafiee Alavi SN, Abbasi MH, et al Comparison of Doppler Ultrasound and Digital Subtraction Angiography in extracranial stenosis. Ann Med Surg (Lond). 2022;74:103202. 35070286 10.1016/j.amsu.2021.103202 PMC8761599

[14] Psychogios K , Magoufis G, Kargiotis O, et al Ultrasound assessment of extracranial carotids and vertebral arteries in acute cerebral ischemia. Medicina (Kaunas). 2020;56:711. 33353035 10.3390/medicina56120711 PMC7765801

[15] Lee W. General principles of carotid Doppler ultrasonography. Ultrasonography. 2014;33:11-17. 24936490 10.14366/usg.13018 PMC4058969

[16] Grant EG , Benson CB, Moneta GL, et al Carotid artery stenosis: gray-scale and Doppler US diagnosis—Society of Radiologists in Ultrasound Consensus Conference. Radiology. 2003;229:340-346. 14500855 10.1148/radiol.2292030516

[17] Oates CP , Naylor AR, Hartshorne T, et al Joint recommendations for reporting carotid ultrasound investigations in the United Kingdom. Eur J Vasc Endovasc Surg. 2009;37:251-261. 19046904 10.1016/j.ejvs.2008.10.015

[18] Calabia J , Torguet P, Garcia I, et al The relationship between renal resistive index, arterial stiffness, and atherosclerotic burden: the link between macrocirculation and microcirculation. J Clin Hypertens (Greenwich). 2014;16:186-191. 24548343 10.1111/jch.12248 PMC8031534

[19] Vengamma B , Challa SN, Devi BV, Prasad SV, Pynam P, Reddy R. Assessment of intracranial and extracranial atherosclerosis in patients presenting with acute ischaemic stroke. J Clin Sci Res. 2020;9:155-159.

[20] Vajpeyee A , Tiwari S, Yadav LB, Gupta S, Vajpeyee M. Pattern of intracranial versus extracranial atherosclerotic cerebrovascular disease in Indian patients with stroke: an angiography study. IJN. 2020;6:183-187.

[21] Wang D , Wang J, Jin C, et al Asymptomatic extracranial artery stenosis and the risk of cardiovascular and cerebrovascular diseases. Sci Rep. 2016;6:33960. 27650877 10.1038/srep33960 PMC5030632

[22] Owolabi MO , Thrift AG, Mahal A, et al; Stroke Experts Collaboration Group. Primary stroke prevention worldwide: translating evidence into action. Lancet Public Health. 2022;7:e74-e85. 34756176 10.1016/S2468-2667(21)00230-9 PMC8727355

[23] Fonarow GC , Reeves MJ, Zhao X, et al; Get With the Guidelines-Stroke Steering Committee and Investigators. Age-related differences in characteristics, performance measures, treatment trends, and outcomes in patients with ischemic stroke. Circulation. 2010;121:879-891. 20142445 10.1161/CIRCULATIONAHA.109.892497

[24] Viswanathan V , Jamthikar AD, Gupta D, et al Does the carotid bulb offer a better 10-year CVD/stroke risk assessment compared to the common carotid artery? A 1516 ultrasound scan study. Angiology. 2020;71:920-933. 32696658 10.1177/0003319720941730

[25] Maitas O , Bob-Manuel T, Price J, et al Vertebral artery interventions: a comprehensive updated review. Curr Cardiol Rev. 2023;19:e170322202296. 35301953 10.2174/1573403X18666220317093131 PMC10201878

[26] Saligommula R , Alam KC. Clinical study of 2D Echo cardiography findings in stroke- ischemic patients. Int J Adv Med. 2019;7:128.

[27] Yoon HJ , Kim KH, Park H, et al Carotid plaque rather than intima-media thickness as a predictor of recurrent vascular events in patients with acute ischemic stroke. Cardiovasc Ultrasound. 2017;15:19-18. 28738808 10.1186/s12947-017-0110-y PMC5525267

[28] Johnsen SH , Mathiesen EB. Carotid plaque compared with intima-media thickness as a predictor of coronary and cerebrovascular disease. Curr Cardiol Rep. 2009;11:21-27. 19091171 10.1007/s11886-009-0004-1

[29] Kwah LK , Diong J. National Institutes of Health Stroke Scale (NIHSS). J Physiother. 2014;60:61. 24856948 10.1016/j.jphys.2013.12.012

[30] Beach KW , Leotta DF, Zierler RE. Carotid Doppler velocity measurements and anatomic stenosis: correlation is futile. Vasc Endovascular Surg. 2012;46:466-474. 22786979 10.1177/1538574412452159

[31] Hallerstam S , Rosfors S. Blood flow and flow resistance in the vertebral arteries of patients with and without carotid atherosclerosis. Clin Physiol Funct Imaging. 2004;24:96-102. 15056182 10.1111/j.1475-097X.2004.00536.x

[32] Lin R , Chen S, Liu G, Xue Y, Zhao X. Association between carotid atherosclerotic plaque calcification and intraplaque hemorrhage: a magnetic resonance imaging study. Arterioscler Thromb Vasc Biol. 2017;37:1228-1233. 28450297 10.1161/ATVBAHA.116.308360

[33] Baradaran H , Al-Dasuqi K, Knight-Greenfield A, et al Association between carotid plaque features on CTA and cerebrovascular ischemia: a systematic review and meta-analysis. AJNR Am J Neuroradiol. 2017;38:2321-2326. 29074638 10.3174/ajnr.A5436 PMC7963758

[34] Wardlaw JM , Stevenson MD, Chappell F, et al Carotid artery imaging for secondary stroke prevention: both imaging modality and rapid access to imaging are important. Stroke. 2009;40:3511-3517. 19729602 10.1161/STROKEAHA.109.557017

[35] Zavanone C , Ragone E, Samson Y. Concordance rates of Doppler ultrasound and CT angiography in the grading of carotid artery stenosis: a systematic literature review. J Neurol. 2012;259:1015-1018. 22064974 10.1007/s00415-011-6265-9

[36] Netuka D , Belšán T, Broulíková K, et al Detection of carotid artery stenosis using histological specimens: a comparison of CT angiography, magnetic resonance angiography, digital subtraction angiography and Doppler ultrasonography. Acta Neurochir (Wien). 2016;158:1505-1514. 27255656 10.1007/s00701-016-2842-0

[37] Birmpili P , Porter L, Shaikh U, Torella F. Comparison of measurement and grading of carotid stenosis with computed tomography angiography and Doppler ultrasound. Ann Vasc Surg. 2018;51:217-224. 29522870 10.1016/j.avsg.2018.01.102

[38] Boyko M , Kalashyan H, Becher H, et al Comparison of carotid Doppler ultrasound to other angiographic modalities in the measurement of carotid artery stenosis. J Neuroimaging. 2018;28:683-687. 29917285 10.1111/jon.12532

